# Effective coverage of newborn postnatal care: assessing the service contact-content gap in 32 low- and middle-income countries using household survey data

**DOI:** 10.7189/jogh.15.04219

**Published:** 2025-09-01

**Authors:** Safia S Jiwani, Assanatou Bamogo, Elizabeth A Hazel, Abdoulaye Maiga, Emily B Wilson, Gouda Roland Mesmer Mady, Agbessi Amouzou

**Affiliations:** Department of International Health, Johns Hopkins Bloomberg School of Public Health, Baltimore, Maryland, USA

## Abstract

**Background:**

High-quality postnatal care (PNC) is essential for newborn survival. However, newborn PNC coverage indicators do not reflect the quality of care received. We estimated effective coverage of newborn PNC by incorporating content of care and calculated the contact-content gap in 32 low-and middle-income countries (LMICs).

**Methods:**

Using household survey data from 32 LMICs, we defined effective coverage of newborn PNC as the proportion of mothers or babies who received five essential signal functions as part of their newborn’s PNC check by a medically trained provider within the first two days of birth. We calculated the contact-content gap as the absolute difference between service coverage of newborn PNC and effective coverage of newborn PNC, in percentage points (pp). We described inequalities in effective coverage of newborn PNC by mother’s age, parity, wealth, education, residence, antenatal care seeking, delivery facility type, and managing authority.

**Results:**

The median effective coverage of newborn PNC was 27.5% across countries, ranging from 2.1% (95% confidence interval (CI) = 1.6, 2.7) in Burundi to 78.3% (95% CI = 74.3, 81.8) in Armenia. We identified large PNC contact-content gaps, up to 52.6 pp in The Gambia. Content of care was generally poor, with only 37.1% of mothers across countries receiving counselling on newborn danger signs. We found important demographic, socioeconomic, and health systems inequalities in newborn PNC effective coverage, disproportionately favouring newborns whose mother was older (35–49 years), primiparous, lived in urban areas, belonged to the wealthiest households, had at least secondary education, and delivered in a hospital or the private sector in most countries.

**Conclusions:**

In most countries, a substantial number of newborns who are checked during PNC do not receive all basic services. Effective coverage measures offer a more comprehensive estimate for assessing service gaps, driving action, and ensuring health gains. Addressing the missed opportunities of inadequate care content and focussing on equity will be critical to improve survival of all mothers and newborns.

In 2022, approximately 2.3 million newborns around the world died within the first month of life [1], with the leading causes being preterm births, birth asphyxia, and neonatal infections [1]. Most newborn deaths occur in low- and middle-income countries (LMICs) and can be prevented by ensuring high-quality, integrated, and holistic intrapartum and postnatal care (PNC) [1–3]. However, while there has been marked progress in the coverage of life-saving interventions such as institutional deliveries [4,5], the quality of care often varies, and few women remain in the facility for the recommended 24 hours to monitor for complications [1].

The postnatal period covers the time immediately following childbirth and up to 42 days after. During this time, the World Health Organization recommends four newborn PNC visits, with the first 24 hours after birth being a critical period for screening and monitoring maternal and newborn health [6]. Newborn PNC is an essential component of the continuum of maternal and newborn care, covering life-saving interventions to detect complications that may have occurred during pregnancy and childbirth, as well as preventing those that may arise postpartum [1,6]. However, maternal and newborn health monitoring has largely focussed on the recommended number and timing of PNC visits, with less emphasis placed on the content of these visits [2]. To address this gap, McCauley and colleagues proposed in their 2022 systematic review the development of 14 signal functions reflecting essential components of PNC and addressing the major causes of maternal and newborn morbidity and mortality [2]. For the newborn baby, the essential signal functions included hygienic cord care, monitoring of growth and development, screening for congenital diseases, counselling and support for exclusive breastfeeding, assessing and counselling for newborn danger signs, and immunisations to prevent infections [2,6].

Globally, only 64% of newborns received a newborn PNC visit within two days of birth between 2014 and 2018 [7]. However, these measures of health intervention coverage, defined as the proportion of the population in need of a health service who receive it, do not capture the content or the quality of care, beyond mere contact with health facilities [8]. Amouzou and colleagues suggest that, despite improved utilisation of maternal and newborn health services in the last decades, progress in maternal and child survival is falling short [9]. This pattern indicates that important gaps exist between contact with health facilities (service coverage) and the gains from these health services in LMICs.

To this end, effective service coverage measures can be used to assess the proportion of individuals who receive quality care to satisfy their specific service needs [10] and have been used to reflect aspects of serving need, service use, and service quality [8,10]. In particular, these include quality-adjusted coverage measures, which can be estimated by linking household survey data with health facility assessment data and adjusting service coverage with measures of quality of care or service readiness [11,12]. However, such linking analyses require temporally aligned household and health facility surveys. Moreover, they are constrained by the inconsistent use of survey tools across countries, limiting the comparability of estimates. In the absence of linked data, some authors have estimated effective coverage of health services using household survey data solely, while incorporating measures of self-reported content of care [13]. Here, we followed this approach and used household survey data from 32 LMICs to estimate effective coverage of newborn PNC services; quantify the service contact-content gap in newborn PNC; and uncover socioeconomic, demographic, and health systems inequalities in effective coverage of newborn PNC.

## METHODS

### Study design and data collection

In this cross-sectional study, we used household surveys from 32 LMICs with available data on PNC content, located in sub-Saharan Africa, Eastern Europe and Central Asia, South Asia, East Asia and Pacific, the Caribbean, and the Middle East. We accessed the latest Demographic and Health Surveys (DHSs) [14] since 2015, from each country with available data pertaining to women’s reports of newborn PNC content and timing, for their most recent birth. The DHSs are nationally representative household surveys with a multistage sampling design conducted in over 90 LMICs and covering key reproductive, maternal, newborn, and child health indicators [14].

### Measures

We defined newborn PNC service coverage as the proportion of women with a live birth in the two years preceding the survey whose newborns received a PNC check by a medically trained provider within the first two days of birth. Effective newborn PNC coverage was defined as the proportion of women with a live birth two years preceding the survey whose newborns received the following five essential signal functions as part of their PNC check by a medically trained provider within first two days of birth (as reported by the women): newborn’s umbilical cord was checked, newborn’s temperature was measured, women received counselling on newborn danger signs, women received counselling on breastfeeding, and breastfeeding was observed. We used these five signal functions as they were the only ones collected uniformly across countries as part of the newborn PNC module in the DHS questionnaire, and these questions included timing of their receipt (within two days of birth). Medically trained providers were defined as doctors, nurses, midwives, and any other country-specific trained cadres specified in the country’s corresponding DHS report [14]. The coverage-content gap was then computed as the absolute difference between newborn PNC coverage and newborn PNC effective coverage, in percentage points (pp).

### Statistical analysis

We restricted the analysis to women of reproductive age (15–49 years) who gave birth two years preceding the survey. We excluded women who experienced an early neonatal death within two days of birth. We reported service coverage and effective coverage of newborn PNC in percentages, along with corresponding 95% confidence intervals (CIs). We also disaggregated PNC effective coverage by demographic factors such as women’s age (15–19 years, 20–34 years, 35–49 years) and parity (primiparous, multiparous); socioeconomic factors such as their place of residence (urban, rural), household wealth status (richest quintile, poorest quintile), their highest education level (no formal education, primary, secondary or more); as well as health systems and care seeking factors such as the facility type of birth (hospital, health centre/other), facility managing authority (public, private) and four or more antenatal care seeking visits (ANC4+) (<4 ANC, 4+ ANC). We generated equiplots to visualise these inequalities across countries. All analyses took into account the surveys’ multistage cluster design and sampling weights and were conducted using Stata, version 17 (StataCorp LLC, College Station, TX, USA).

## RESULTS

### Newborn PNC coverage

We included 118 424 newborns from 32 countries in this analysis, ranging from 672 in Armenia (2016) to 12 366 in Nigeria (2018). The median service coverage of newborn PNC across all countries was 66.5%, reflecting the proportion of women whose newborn received a PNC check by a skilled provider within two days of birth. There were wide variations in service coverage across countries, ranging from 20.6% (95%CI = 18.8, 22.5) in Angola (2015) to 98.3% (95% CI = 96.8, 99.1) in Armenia (2016) (Tables S1 and S2 in **Online Supplementary Document**).

### Newborn PNC effective coverage

Effective coverage of newborn PNC reflects the receipt of five signal functions as part of PNC, beyond the contact with the provider. Our estimates for this were much lower than those for service coverage, with a median of 27.5% across countries. The lowest was in Burundi (2017), where only 2.1% (95% CI = 1.6, 2.7) of women’s newborns received effective PNC, and the highest was in Armenia (2016), where 78.3% (95% CI = 74.3, 81.8) of them received effective PNC. Overall, Armenia, South Africa, and Tajikistan achieved the highest newborn PNC effective coverage, while Angola, Burundi, Ethiopia, and Timor Leste were in the lowest rank across all 32 countries (Tables S1 and S2 in **Online Supplementary Document**).

Focussing on the coverage of the specific signal functions, less than half of women reported that their newborn received an umbilical cord check and a temperature measurement during PNC, with medians across countries of 46.5% and 46.3%, respectively. While in some countries, such as Armenia, over 80% of women reported their newborns received these five key signal functions, the majority of them reported lower coverage at below 50% (Tables S1 and S2 in **Online Supplementary Document**). In Burundi, for instance, only 5.3% (95% CI = 4.5, 6.1) of women reported their newborn’s umbilical cord was checked, while 4.4% (95% CI = 3.8, 5.2) had their temperature measured. Moreover, coverage of counselling interventions such as women’s counselling on newborn danger signs, counselling of breastfeeding, and observation of breastfeeding had the lowest coverage, with large variability across countries. For example, counselling on newborn danger signs was received by only 3.5% (95% CI = 2.9, 4.2) of women in Burundi, compared to 86.3% (95% CI = 82.5, 89.4) in Armenia, with a median of 37.1% across all countries. Similarly, observation of breastfeeding across countries was the lowest signal function, with a median coverage of 36.9% (Table S1 in **Online Supplementary Document**).

### Newborn PNC contact-content gap

**Figure 1:**
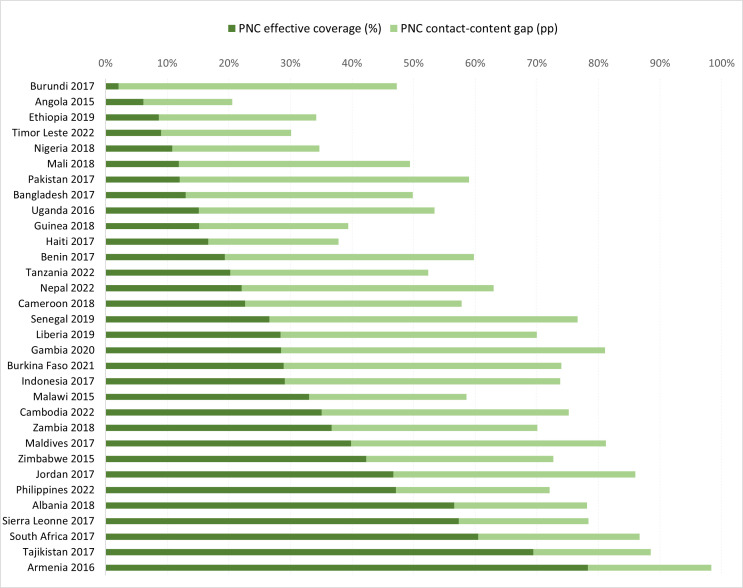
Contact-content gap in newborn PNC, by country.

The lower effective coverage estimates compared to service coverage estimates for newborn PNC indicate important contact-content absolute coverage gaps. While service coverage measures in The Gambia suggested that 81.1% (95% CI = 79.1, 82.9) of women received a newborn PNC check by a skilled provider within two days of birth, only 28.5% (95% CI = 25.7, 31.4) of them received all five signal functions as part of that PNC check, denoting the largest contact-content gap of 52.6 pp. Only three countries (Angola, Armenia, Tajikistan) had a content-contact gap of 20 pp or less. Notably, the small absolute gap in Angola can be misleading, as it stemmed from poor PNC coverage (20.6%) and poor effective coverage (6.1%); in this case, the difference can better be expressed as a relative gap, where PNC coverage was 3.4 times that of PNC effective coverage. In contrast, in Armenia and Tajikistan, the absolute content-contact gap reflected a small difference between equally high service coverage and effective coverage of newborn PNC. Additionally, 18 countries had an absolute gap between 20 pp and 40 pp, and 11 countries had a gap larger than 40 pp (Benin, Burkina Faso, Burundi, Cambodia, Indonesia, Liberia, Maldives, Nepal, Pakistan, Senegal, The Gambia) (**Figure 1**; Table S1 in **Online Supplementary Document**).

### Inequalities in PNC effective coverage

#### Demographic inequalities

**Figure 2:**
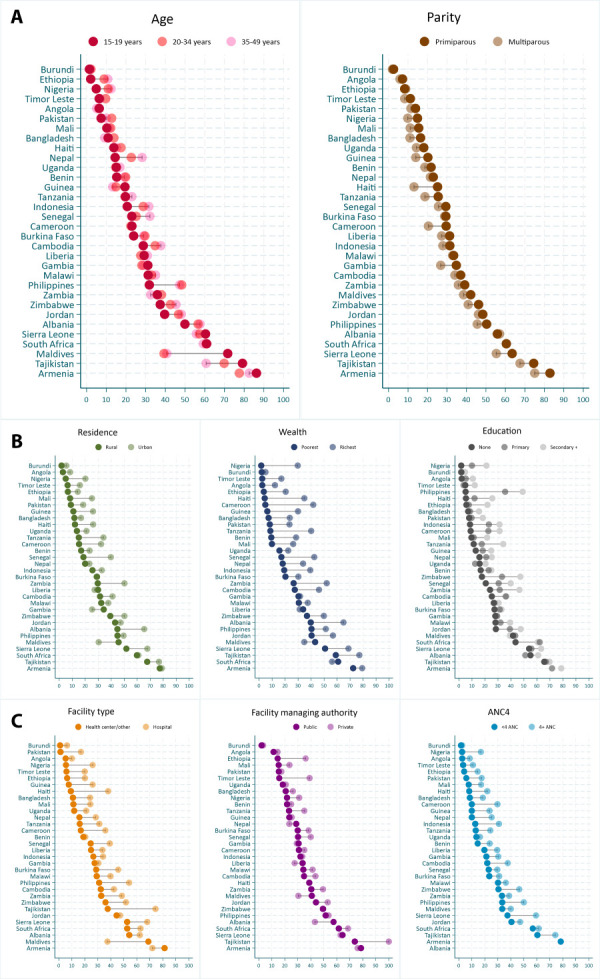
Panels A–C. **Panel A.** Demographic inequalities in newborn PNC effective coverage (%), by country. **Panel B.** Socioeconomic inequalities in newborn PNC effective coverage (%), by country. **Panel C.** Health systems and care seeking inequalities in newborn PNC effective coverage (%), by country.

Disaggregating newborn PNC effective coverage by mothers’ key characteristics reveals important differences between subgroups. In terms of demographic factors such as mothers’ age and parity, we observed the lowest PNC effective coverage among women aged 15–19 years in most countries, except for Zambia, Sierra Leone, Guinea, the Maldives, South Africa, and Armenia. The largest inequalities in favour of younger women are observed in the Maldives, where 71.7% of women aged 15–19 years received effective newborn PNC compared to 40.7% of those aged 35–49 years. Armenia has the highest effective PNC coverage among all age groups, particularly among younger women (86.3%), whereas Burundi has the lowest, with only 1.4% and 2.0% of women aged 15–19 years and 35–49 years, respectively, receiving effective newborn PNC (**Figure 2**, Panel A).

In all countries except Albania, more primiparous women received effective PNC compared to multiparous women, with the largest difference observed in Haiti (25.2% of primiparous women and 13.1% of multiparous women). Armenia and Burundi remain the countries with the highest and lowest effective coverage estimates, respectively, for each age and parity subgroup (**Figure 2**, Panel A).

### Socioeconomic inequalities

Residence, wealth, and education showed similar equity patterns, with much wider differences by wealth and education. In most countries, effective PNC coverage was higher among urban women, those living in the wealthiest households, and those with secondary education or higher. However, the reverse was true in Liberia (favouring rural and poorest women), The Gambia and the Maldives (favouring rural women), and South Africa (favouring the poorest women).

We observed the widest inequalities by wealth in Cameroon, where only 18.1% of women from the poorest households received effective newborn PNC, compared to 41.6% of the richest households. This was also the case in the Philippines by education, where effective coverage was 5.1% among mothers with no formal education and 49.0% among mothers with at least secondary level education – a large absolute gap of 43.9 pp. While rural mothers in Burundi had the lowest effective coverage estimates by residence, the poorest mothers in Nigeria and those with no formal education had the lowest estimates by wealth and education. The highest effective coverage estimates prevailed in Armenia for each subgroup (**Figure 2**, Panel B)

### Health systems and care-seeking inequalities

Fewer women giving birth in lower-level facilities such as health centres and the private sector received effective PNC compared to their counterparts in hospitals and the public sector. Large differences by facility type were found in Tajikistan, Haiti, the Philippines and Nigeria, as well as by facility managing authority observed in Ethiopia, Timor Leste and Tajikistan, favouring private facility and hospital births. For example, in Tajikistan, the effective PNC coverage estimates among newborns in health centres were 37.6%, compared to 74.3% among those born in hospitals, indicating a 36.7 pp absolute gap. All births from the private sector received effective PNC, compared to 73.8% in the public sector.

However, the Maldives and Armenia displayed the opposite pattern of improved newborn PNC effective coverage among women delivering in health centres, with absolute inequality gaps as large as 31.5 pp in the Maldives. Similarly, in Nepal, The Gambia, Liberia, Sierra Leone, the Maldives, Albania, and Armenia, we estimated a higher effective PNC coverage among births in the public sector compared to the private sector.

In all countries, women who received 4 or more ANC visits had higher effective coverage of newborn PNC than their counterparts who had fewer than 4ANC visits during pregnancy, with inequality gaps as large as 22 pp in Sierra Leone (**Figure 2**, Panel C).

## DISCUSSION

This work builds on existing evidence and pushes towards accounting for the content and quality of the care received by women and children using effective coverage measures, rather than merely measuring service coverage [8,10]. Using household survey data since 2015, we estimated a median newborn PNC effective coverage of 27.5% across 32 LMICs, with wide variability across countries, ranging from the lowest estimate of 2.1% (95% CI = 1.6, 2.7) in Burundi to the highest estimate of 78.3% (95% CI = 74.3, 81.8) in Armenia. These estimates, which account for the receipt of five key signal functions during the PNC visit, are much lower than those obtained by merely measuring newborn PNC service coverage, which were already suboptimal in most countries (median of 66.5%).

In fact, we calculated a large median contact-content PNC gap of 34.3 pp across countries, and as large as 52.6 pp in The Gambia. This gap was smaller in countries with low or high PNC service coverage, compared to that in countries with medium PNC service coverage (between 40% and 80%). We identified two countries (Armenia and Tajikistan), where the newborn PNC contact-content gap was 20 pp or below and was driven by both high service coverage and high effective coverage of newborn PNC, pointing to high access and utilisation, as well as the content and presumably the quality of the service. In Angola specifically, the 14.5 pp gap stemmed from poor service coverage, reflecting challenges in access and utilisation, in addition to poor quality of care. Conversely, we identified 11 countries which had a contact-content gap above 40 pp, driven by much lower effective coverage compared to service coverage (Benin, Burkina Faso, Burundi, Cambodia, Indonesia, Liberia, Maldives, Nepal, Pakistan, Senegal, The Gambia).

Other studies have estimated effective coverage of newborn PNC, though the majority lacked multi-country comparisons. Gebremedhin and colleagues used DHS surveys in Ethiopia from 2016 to estimate an effective newborn PNC coverage of 13.2%, compared to our estimate of 8.6% in 2019 [15], with key differences in the definitions used to estimate effective coverage. For instance, the type of provider was not accounted for in their definition, whereas we included PNC visits from a skilled provider. Moreover, the authors defined quality based on receipt of at least five out of 11 possible signal functions, whereas we defined it as receiving all five signal functions [15]. Our more stringent definition of effective coverage measures, taking into account key elements of quality care (including the type of provider and the receipt of all five signal functions), helps explain the lower estimate in our analysis. Due to the lack of standardised definitions for computing and assessing the effective coverage of services, drawing comparisons across studies becomes a challenging endeavour.

Specifically, our findings highlight that, while a newborn may be checked by a skilled provider within two days of birth, that does not imply a complete PNC assessment and the health gains that should stem from it. We found that only a median of 37.1% of mothers across countries received counselling on newborn danger signs, while only 36.9% had a breastfeeding observation. These interventions are critical for preventing health complications that may arise in the postnatal period and for promoting healthy newborn growth and development [1,2]. More research is needed to identify health systems and provider-level barriers in conducting such interventions during PNC. Studies measuring facility readiness for maternal and newborn health services have pointed to key structural gaps in physical resources (equipment, supplies and medicines), as well as systemic challenges such as insufficient trained health workers to meet high-quality standards of care [16–19]. For instance, Niehaus and colleagues identified important barriers in the availability of basic supplies for small and/or sick newborn care, such as thermometers for low-body temperature and infant and young child feeding guidelines in Malawi, Mozambique and Tanzania. Similarly, only 29% of staff in service were trained on kangaroo mother care and less than half were trained in hygienic cord care and thermal care [19]. Other studies have found better facility readiness for maternal and newborn health services in hospital settings compared to lower-level facilities [19–22].

Simultaneously, it is essential to implement comprehensive policies that not only support healthcare providers, but also empower them to deliver high-quality PNC. These policies should aim to create a robust framework that ensures providers have the necessary resources, training, and incentives to offer effective, compassionate, and accessible care to mothers and their newborns. By fostering an environment where providers are well-equipped and motivated, governments can improve the quality of PNC and promote the health and well-being of both mother and child [23,24].

National-level estimates often hide inequities that persist in accessing and receiving high-quality newborn PNC services. We observed important demographic, socioeconomic, and health systems gaps in effective newborn PNC coverage estimates, suggesting that, in most countries, high content newborn PNC was disproportionately in favour of newborns whose mothers were older (35–49 years), primiparous, lived in urban areas, belonged to the wealthiest households, had at least secondary education, and delivered in a hospital or the private sector. Similar patterns were reported by Kim and colleagues' equity analysis of maternal PNC effective coverage in Cambodia, with higher effective coverage of maternal PNC among urban women, those belonging to the highest wealth quintile, and those with secondary education [25]. They also found changing patterns of inequality over time, with a reducing gap between the poorest/richest and rural/urban women [25]. Furthermore, several studies in Ethiopia have pointed to socioeconomic inequities with higher PNC coverage among richer women [26], including Gebremedhin and colleagues, suggesting that effective coverage of newborn PNC was ‛pro-rich’ with a concentration index of 0.439, as well as large gaps between regions in Ethiopia [15]. These inequities may reflect cultural, structural and systemic barriers in accessing high-quality care among poorer women, those living in rural areas and with low education. For instance, they may be influenced by delays in seeking care or receiving care in lower-level and poorly equipped facilities with insufficiently trained health workers.

We observed the opposite patterns in countries such as the Maldives, Armenia, Liberia, South Africa, Sierra Leone, and the Gambia. For instance, in the Maldives, newborns from the poorest households, and those from adolescent mothers had better PNC effective coverage, compared to their richest and older counterparts, respectively; in the Gambia, rural women had higher effective coverage estimates than their urban counterparts. This may be influenced by programmes and interventions focussing on the most vulnerable populations, often targeting rural areas and adolescent women, as well as larger efforts to improve quality of care in facilities serving these marginalised populations. In some countries, this pattern may be related to data or measurement issues, requiring further investigation. The Maldives’ context is unique, with low poverty rates of 1.7% based on the international poverty line, and significant improvements made in the past years to increase access to healthcare, education, and quality of life, especially in atolls – rural areas housing 93% of the country’s low-income population [27]. According to the DHS report, 96.8% of women from the poorest wealth quintile gave birth in a health facility, compared to 88.8% in the richest wealth quintile. Similarly, a higher proportion of women from the richest quintile, and living in the capital city of Male, reported difficulties in accessing health services when sick, compared to those in the poorest quintile, and living in rural atolls [28]. The inequality patterns observed in the Maldives are therefore a reflection of its unique economic circumstances, health systems, and geographic setting. That said, further research is needed to better understand these inequities, the barriers and facilitators to accessing high-quality care, and, importantly, to identify drivers of effective newborn PNC.

The main strength of our study was the use of data from 32 LMICs, which addressed the dearth of scientific evidence on effective coverage estimates of PNC, and highlighted the persisting inequities by demographic, socioeconomic, and health systems dimensions. However, there were also some limitations, such as indicators that were based on the mothers’ recall; evidence suggest that women don’t always accurately recall interventions received around the time of childbirth [29]. Moreover, recall for specific interventions may vary by women’s socioeconomic status [30]. We minimised this issue by restricting the sample to women who gave birth two years before the survey. Furthermore, in some settings, newborns may be taken to a separate room for PNC examinations, affecting mothers’ responses to care content. Additionally, we did not include the full set of signal functions of newborn PNC, as we were limited by variables captured in the DHS questionnaires across all 32 countries, and we focussed our analysis on the signal functions captured within the PNC module that referred to being conducted within two days of birth. Our data covered a wide range of survey years from 2015 to 2022 across countries; these temporal differences were not adjusted and may reflect different healthcare contexts, particularly during the COVID-19 pandemic. Moreover, wealth quintiles across countries reflect different socioeconomic realities; therefore, comparisons across countries ought to be interpreted with caution. Lastly, we excluded newborns who died within the first two days, as we did not know whether they would have received the PNC signal functions should they have survived; this could lead to an overestimation of the effective coverage measure if the lack of PNC was the driving factor of the death within the first two days.

## CONCLUSIONS

Our analysis of household survey data from 32 LMICs underscores the significant contact-content gaps in newborn PNC. Effective coverage measures incorporating content/quality of care offer a more comprehensive estimate to drive action and ensure gains from health services; these measures ought to be used more widely to monitor maternal and newborn health globally. However, efforts are needed to standardise definitions and allow comparisons across studies. Our study highlights the need for policies and improvements in health systems to overcome barriers that hinder effective PNC. While progress has been made in increasing access to care, the content of care remains inadequate and incomplete, and effective PNC disproportionately favours the wealthy, urban, and educated populations in most countries. Concretely, multi-component interventions aiming to enhance health worker training and service readiness in lower-level facilities, as well as to strengthen service delivery through standardised protocols, monitoring and quality assurance mechanisms, are critical towards improving effective coverage of newborn PNC. Addressing these missed opportunities and large contact-content gaps in PNC, focussing on equity, and strengthening health systems will be key to ensuring the survival of all mothers and newborns.

## Additional material


Online Supplementary Document
