# Supplementary material for: Effective coverage of newborn postnatal care: assessing the service contact-content gap in 32 low- and middle-income countries using household survey data
Source: J Glob Health. 2025 Sep 1;15:04219. doi: 10.7189/jogh.15.04219 (PMC12404218; doi:10.7189/jogh.15.04219)
Supplement: Online Supplementary Document [file jogh-15-04219-s001.pdf]

Supplement to: Jiwani SS, Bamogo A, Hazel EA, Maiga A, Wilson EB, Mady GRM, Amouzou A. Effective coverage of newborn postnatal care: assessing the service contact-content gap in 32 low- and middle-income countries using household survey data. J Glob Health. 2025;15:04219.

Table 1S. newborn PNC coverage, effective coverage, and signal function coverage by country (%).

Country, survey year	Sample of newborns (N)	Newborn PNC Service coverage		Newborn PNC Effective coverage		Cord check		Temperature measurement		Counseling: danger signs		Counseling: breastfeeding		Observation breastfeeding	
		%	95% CI	%	95% CI	%	95% CI	%	95% CI	%	95% CI	%	95% CI	%	95% CI
Albania, 2017-18	1,036	78.14	73.94, 81.82	56.58	51.89, 61.15	70.72	66.43, 74.67	71.55	67.38, 75.39	63.61	59.23, 67.78	67.67	63.14, 71.88	65.35	60.86, 69.57
Angola, 2015	5,647	20.57	18.77, 22.49	6.15	5.05, 7.46	10.67	9.22, 12.33	10.56	9.14, 12.18	10.49	9.09, 12.08	11.83	10.27, 13.60	10.00	8.57, 11.66
Armenia, 2016	672	98.31	96.80, 99.11	78.31	74.28, 81.86	97.80	96.01, 98.80	96.33	94.22, 97.69	86.28	82.50, 89.35	94.26	91.74, 96.04	87.84	84.31, 90.67
Bangladesh, 2017	3,377	49.86	47.32, 52.41	13.02	11.54, 14.65	46.27	43.76, 48.81	47.08	44.61, 49.57	20.83	18.99, 22.79	44.06	41.55, 46.59	29.69	27.53, 31.94
Benin, 2017	5,309	59.77	57.49, 62.01	19.36	17.77, 21.06	33.71	31.70, 35.79	35.14	33.16, 37.18	28.11	26.30, 29.10	32.80	30.91, 34.74	28.18	26.34, 30.08
Burkina Faso, 2021	4,571	74.02	71.91, 76.02	28.93	26.77, 31.19	39.68	37.36, 42.05	39.95	37.62, 42.32	34.24	31.97, 36.57	38.99	36.79, 41.23	35.51	33.33, 37.75
Burundi, 2017	5,159	47.29	45.39, 49.19	2.11	1.64, 2.69	5.25	4.51, 6.11	4.44	3.81, 5.17	3.52	2.93, 4.22	4.98	4.31, 5.74	4.16	3.55, 4.88
Cambodia, 2022	3,307	75.21	72.94, 77.35	35.07	32.66, 37.55	60.48	58.06, 62.85	50.14	47.68, 52.60	50.30	47.84, 52.75	56.91	54.28, 59.51	47.98	45.45, 50.52
Cameroon, 2018	3,670	57.78	54.63, 60.87	22.64	20.57, 24.85	38.95	36.07, 41.91	41.40	38.49, 44.38	32.29	29.88, 34.80	36.29	33.67, 38.99	31.42	28.99, 33.95
Ethiopia, 2019	2,063	34.19	29.73, 38.95	8.67	6.66, 11.22	19.07	15.95, 22.64	18.67	15.58, 22.22	14.70	11.70, 18.30	22.30	19.02, 25.97	21.65	18.06, 25.72
Gambia, 2020	3,335	81.06	79.11, 82.86	28.48	25.72, 31.41	59.35	55.55, 63.03	58.83	55.08, 62.49	43.25	40.12, 46.43	43.25	40.06, 46.49	38.11	35.19, 41.11
Guinea, 2018	2,969	39.43	36.55, 42.39	15.19	13.23, 17.39	23.33	21.04, 25.79	22.98	20.70, 25.45	22.85	20.62, 25.23	22.75	20.47, 25.21	17.99	15.94, 20.25
Haiti, 2017	2,462	37.80	34.80, 40.90	16.69	14.53, 19.11	34.73	31.85, 37.73	34.28	31.41, 37.26	26.69	24.06, 29.47	27.90	25.30, 30.66	19.12	16.83, 21.64

Indonesia, 2017	6,854	73.77	72.17, 75.30	29.08	27.61, 30.59	59.16	57.43, 60.85	50.69	48.98, 52.41	40.31	38.66, 41.97	48.08	46.41, 49.75	42.69	41.08, 44.32
Jordan, 2017	3,885	85.95	83.10, 87.70	46.72	43.61, 49.85	74.56	71.95, 76.10	76.89	74.52, 79.10	62.90	60.09, 65.62	58.84	55.90, 61.72	55.27	52.30, 58.20
Liberia, 2019	2,196	69.95	66.55, 73.14	28.43	25.57, 31.46	41.13	38.00, 44.33	38.28	34.89, 41.78	40.03	36.77, 43.38	43.36	40.09, 46.69	40.34	37.12, 43.65
Malawi, 2015	6,434	58.59	56.86, 60.30	33.06	31.34, 34.82	43.99	42.14, 45.85	47.17	45.34, 49.01	48.42	46.62, 50.22	54.78	53.01, 56.53	47.66	45.76, 49.57
Maldives, 2017	1,135	81.23	77.61, 84.39	39.86	35.90, 43.95	76.91	72.81, 80.56	75.76	71.70, 79.40	49.83	45.31, 54.36	62.02	58.15, 65.74	60.86	56.90, 64.68
Mali, 2018	3,812	49.39	46.13, 52.66	11.89	10.15, 13.88	19.57	17.19, 22.20	18.71	16.27, 21.42	18.05	15.84, 20.50	21.53	18.10, 24.29	20.05	17.64, 22.69
Nepal, 2022	1,948	63.03	60.20, 65.78	22.08	19.64, 24.72	46.68	43.73, 49.65	46.35	43.38, 49.35	32.82	30.07, 35.70	49.83	47.08, 52.58	46.91	44.15, 49.69
Nigeria, 2018	12,366	34.69	33.12, 36.29	10.81	9.93, 11.76	16.42	15.34, 17.55	16.67	15.58, 17.82	14.94	13.95, 15.99	16.20	15.12, 17.34	13.83	12.81, 14.92
Pakistan, 2017	4,582	59.04	55.87, 62.15	12.05	10.46, 13.84	49.99	46.75, 53.23	41.91	38.82, 45.06	24.21	21.77, 26.83	36.69	33.54, 39.95	21.04	18.88, 23.38
Philippines, 2022	2,978	72.07	69.59, 74.43	47.14	44.50, 49.79	61.63	59.01, 64.19	62.20	59.57, 64.76	59.42	56.74, 62.05	59.63	56.97, 62.23	52.59	49.90, 55.25
Senegal, 2019	2,399	76.63	73.02, 79.88	26.59	23.57, 29.85	59.54	55.37, 63.57	58.81	54.66, 62.84	41.88	38.03, 45.83	43.89	40.51, 47.34	34.20	30.84, 37.72
Sierra Leone, 2017	3,838	78.36	76.11, 80.45	57.32	54.62, 59.97	67.75	65.17, 70.23	67.42	64.82, 69.92	66.55	63.93, 69.07	67.96	65.41, 70.41	62.24	59.66, 64.75
South Africa, 2017	1,320	86.74	83.99,89.08	60.50	56.90, 63.99	77.78	73.92, 81.20	77.38	73.58, 80.78	67.83	64.02, 71.42	73.32	69.49, 76.82	69.39	65.76, 72.80
Tajikistan, 2017	2,246	88.51	86.53, 90.23	69.42	66.60, 72.10	81.87	79.34, 84.16	82.93	80.69, 84.97	78.38	75.99, 80.60	78.39	76.11, 80.50	75.62	73.07, 78.00
Tanzania, 2022	4,169	52.35	49.89, 54.81	20.24	18.48, 22.12	36.73	34.35, 39.17	26.95	24.93, 29.06	27.16	25.07, 29.36	35.47	33.17, 37.83	35.62	33.29, 38.02
Timor Leste, 2022	2,765	30.06	27.48, 32.78	09.04	07.45, 10.92	12.78	10.85, 14.98	12.86	10.96, 15.03	11.70	09.95, 13.72	15.47	13.46, 17.72	15.98	14.03, 18.13
Uganda, 2016	5,813	53.38	51.16, 55.58	15.15	13.75, 16.67	30.59	28.77, 32.46	23.43	21.79, 25.15	23.69	22.06, 25.41	27.00	25.31, 28.77	27.21	25.49, 29.00
Zambia, 2018	3,817	70.11	67.77, 72.35	36.68	34.10, 39.34	48.35	45.52, 51.18	46.15	43.30, 49.04	43.73	41.07, 46.42	47.57	44.81, 50.34	45.02	42.36, 47.70
Zimbabwe, 2015	2,290	72.66	69.64, 75.48	42.33	39.54, 45.17	62.82	59.73, 65.82	60.52	57.38, 63.58	52.59	49.69, 55.47	63.04	59.92, 66.06	60.61	57.48, 63.66
